# The human plasma-metabolome: Reference values in 800 French healthy volunteers; impact of cholesterol, gender and age

**DOI:** 10.1371/journal.pone.0173615

**Published:** 2017-03-09

**Authors:** Séverine Trabado, Abdallah Al-Salameh, Vincent Croixmarie, Perrine Masson, Emmanuelle Corruble, Bruno Fève, Romain Colle, Laurent Ripoll, Bernard Walther, Claire Boursier-Neyret, Erwan Werner, Laurent Becquemont, Philippe Chanson

**Affiliations:** 1 Assistance Publique-Hôpitaux de Paris, Hôpitaux Universitaires Paris-Sud, Hôpital de Bicêtre, Service de Génétique moléculaire, Pharmacogénétique et Hormonologie, Le Kremlin Bicêtre, France; 2 Inserm U1185, Fac Med Paris Sud, Université Paris-Saclay, Le Kremlin-Bicêtre, France; 3 Assistance Publique-Hôpitaux de Paris, Hôpitaux Universitaires Paris-Sud, Hôpital de Bicêtre, Service d’Endocrinologie et des Maladies de la Reproduction, Le Kremlin Bicêtre, France; 4 Institut de Recherches Internationales Servier, Suresnes, France; 5 Technologie Servier, Orléans, France; 6 Univ Paris Sud, INSERM UMR 1178, Service de Psychiatrie, équipe "Dépression et Antidépresseurs", Hôpital Bicêtre, Assistance Publique Hôpitaux de Paris, Le Kremlin Bicêtre, France; 7 UPMC Univ Paris 06, INSERM UMR S938, Centre de Recherche Saint-Antoine, Hôpital Saint-Antoine, Assistance Publique Hôpitaux de Paris, Paris, France; 8 Département de Pharmacologie, Faculté de médecine Paris-Sud, Université Paris-Sud, UMR 1184, CEA, DSV/iMETI, Division d’Immuno-Virologie, IDMIT, INSERM Centre d’Immunologie des Infections virales et des Maladies Autoimmunes, Assistance Publique–Hôpitaux de Paris, Hôpital Bicêtre, Le Kremlin Bicêtre, France; National Research Council of Italy, ITALY

## Abstract

Metabolomic approaches are increasingly used to identify new disease biomarkers, yet normal values of many plasma metabolites remain poorly defined. The aim of this study was to define the “normal” metabolome in healthy volunteers. We included 800 French volunteers aged between 18 and 86, equally distributed according to sex, free of any medication and considered healthy on the basis of their medical history, clinical examination and standard laboratory tests. We quantified 185 plasma metabolites, including amino acids, biogenic amines, acylcarnitines, phosphatidylcholines, sphingomyelins and hexose, using tandem mass spectrometry with the Biocrates AbsoluteIDQ p180 kit. Principal components analysis was applied to identify the main factors responsible for metabolome variability and orthogonal projection to latent structures analysis was employed to confirm the observed patterns and identify pattern-related metabolites. We established a plasma metabolite reference dataset for 144/185 metabolites. Total blood cholesterol, gender and age were identified as the principal factors explaining metabolome variability. High total blood cholesterol levels were associated with higher plasma sphingomyelins and phosphatidylcholines concentrations. Compared to women, men had higher concentrations of creatinine, branched-chain amino acids and lysophosphatidylcholines, and lower concentrations of sphingomyelins and phosphatidylcholines. Elderly healthy subjects had higher sphingomyelins and phosphatidylcholines plasma levels than young subjects. We established reference human metabolome values in a large and well-defined population of French healthy volunteers. This study provides an essential baseline for defining the “normal” metabolome and its main sources of variation.

## Introduction

Metabolomics is the comprehensive study and analysis of small-molecule metabolites in biological systems. The overall set of these metabolites in a given biological system is defined as the metabolome. The metabolome integrates an individual’s genetic background, aging, lifestyle and environmental factors [1]. As it closely reflects the phenotype, the metabolome can provide important information about the state of a cell, organ, or organism. As in all other “omics”, great strides have been made in recent years in characterizing the human metabolome and human metabolic type (metabotype) variations. Targeted metabolomics measures accurate concentration of a predefined set of metabolites, based on calibration curves, and standards. Untargeted and targeted approaches can be combined [2, 3]. However, it is still difficult to transpose results from one metabolomic study to another lab. In the present work, we determined reference values of plasma metabolome with a reference commercial kit (Biocrates AbsoluteIDQ p180), which is increasingly used in metabolomic studies. This standardized targeted metabolomic assay facilitates the analysis of large-scale patient cohorts and the comparisons of data originating from different studies.

Knowledge of metabolomics can help to identify biomarkers for disease risk determination and management, and researchers are trying to ascertain metabolic profiles (signatures) for different disease states. Clearly, however, accurate reference human metabolome values must first be established. Standardized metabolomics methods have been successfully applied to very large scale population, like the German KORA and the TwinUK cohorts for example [4], using a prior version of Biocrates kit (Biocrates AbsoluteIDQ p150). However, in these two cohorts, recruitment was performed in the general population, lacking a complete and precise clinical and biological healthy status. Very few well-characterized healthy volunteers (HVs) groups have been studied by such approaches: 15 HVs [5], 100 HVs from the European Prospective Investigation into Cancer and Nutrition (EPIC)-Potsdam study [6], 22 HVs [7] and 54 HVs [2]. However, this latest study was the starting point of the human metabolome project, an online database referring the published metabolites quantitative data, whatever the method of quantification [8]. Before drawing conclusion about disease-related metabolic signatures, well-conducted standardized metabolomic studies with adequate sample sizes are needed to obtain reliable reference values of the human metabolome. The present study was thus conducted to edit accurate reference values in a large sample of well-characterized healthy French volunteers (VARIETE study), representing all adult age groups (about 100 subjects per decade) with a balanced sex ratio. Careful exclusion of subjects with medication or intercurrent disease was performed. Compared to previous studies, the present one gets strength from a particularly strict recruitment based on age and sex stratification, as well as clinical and biochemical examination, in order to accurately define healthy volunteers. We used the novel version of Biocrates AbsoluteIDQ p180 kit, which, compared to the previous p150 kit, allows quantitative, precise and reproducible measurement of all twenty-one amino acids and some biogenic amines, in order to establish reference metabolome values. These reference values will be a valuable tool for interpreting values obtained in various pathological situations. In this HVs cohort, the second aim was to determine the main biochemical and physiological main factors responsible for the metabolome variability.

## Subjects and methods

### Subjects and ethical statement

We included healthy volunteers (HVs) who participated in the VARIETE study, a population-based cross-sectional study designed to establish reference values for insulin-like growth factor 1 (IGF-1) in the general population (ClinicalTrials.gov Identifier: NCT01831648) [9]. Subjects were recruited by the clinical research units of 10 French university hospitals and enrolled between January 2011 and February 2012. To be included in the study, subjects aged between 18 and 89 had to be considered healthy, based on their medical history, clinical examination (including nutritional status and gonadal/sexual status), routine laboratory tests after an overnight fast (plasma sodium, potassium, calcium, phosphate, creatinine, glycemia, total blood cholesterol (TBC), liver enzymes, TSH, complete blood count, albuminemia, prothrombin time, and HIV, HBV and HCV serology), and BMI (between 19 and 28 kg/m^2^). The exclusion criteria were a medical history of thyroid, metabolic, endocrine, renal, hepatic, cardiovascular, pulmonary, gastrointestinal or psychiatric disease, chronic infection, cancer or epilepsy; illness during the week preceding inclusion; illicit drug use; use of treatments potentially modifying IGF-I or calcium/phosphorus metabolism (corticosteroids, antiandrogens or antiestrogens, loop diuretics, hydrochlorothiazide, CYP inducers); pregnancy or breast-feeding; and a history of blood transfusion or donation within the 3 months before inclusion. The aim was to recruit 1000 volunteers aged between 18 and 90, with a 1:1 sex ratio in each 10-year age bracket. All the participants gave their written informed consent before entering the study, which was approved by the relevant national authority (l’Agence nationale de sécurité du medicament et des produits de santé) and by the Ile de France VII ethics committee. The VARIETE study was financed by a national grant (PHRC, AOM09122) and sponsored by Assistance Publique-Hôpitaux de Paris (APHP, Paris, France). Metabolomic analyses were performed at Technologie Servier (Orléans, France).

### Sample collection and preparation

Blood samples were obtained between 8:00 and 10:00 a.m. after an overnight fast. In addition to the blood samples necessary for the screening biological evaluation, 30 mL of EDTA blood was obtained from each subject. Blood was centrifuged immediately (10 minutes, 2000 *g* at 4°C) and plasma was aliquoted into separate polypropylene tubes that were immediately stored at -80°C. A 1 mL aliquot of frozen plasma from each subject was sent to Technologie Servier, Orléans, France, on dry ice, and stored at -80°C until analysis. Each aliquot was further divided into the volumes required for different analytical methods. One 10-μL aliquot was analyzed with the Biocrates AbsoluteIDQ p180 kit (Biocrates Life Science AG, Innsbruck, Austria). The plasma samples were processed as recommended by the manufacturer and analyzed on an API 4000 Q-TRAP mass spectrometer (AB Sciex, Darmstadt, Germany) coupled to an ACQUITY UPLC I Class system (Waters Corporation, Milford, MA, USA) equipped with an Agilent C_18_ HPLC column.

### Targeted identification and quantification

The Biocrates AbsoluteIDQ p180 kit allows the identification and measurement of more than 180 metabolites, including amino acids, biogenic amines, acylcarnitines, lysophosphatidylcholines, phosphatidylcholines, sphingomyelins and the sum of hexoses (one resultant metabolite). Amino acids and biogenic amines were analyzed by liquid chromatography (LC) coupled to tandem mass spectrometry, while other metabolites were analyzed using flow injection analysis (FIA) coupled to tandem mass spectrometry. Identification and quantification were performed based on internal standards and multiple reactions monitoring (MRM) detection. After a pre-processing step (peak integration and concentration determination from calibration curves) with Multiquant software (AB Sciex, Darmstadt, Germany), data were uploaded into Biocrates Met*IDQ* software (included in the kit). Concentrations of metabolites monitored by FIA were directly calculated in Met*IDQ*.

### Data management and analysis

Values below the lower limit of quantification (LLOQ) (or below the limit of detection (LOD) for FIA metabolites) were reported as ND (not detected). Values above the upper limit of quantification (ULOQ) were reported as “>” the actual ULOQ. Thus, reported concentrations were within the quantification range validated for each metabolite.

Fourteen p180 kits were necessary to analyze the whole cohort. Data normalization to correct for between-kit variation was based on the median value of Biocrates QC Level 1 on each kit (4 replicates / kit). The LLOQ and ULOQ were normalized accordingly.

### Statistical analysis

Multivariate analyses—principal components analysis (PCA), orthogonal projection to latent structures (OPLS) analysis and OPLS-discriminant analysis (OPLS-DA)–were performed with SIMCA-P v13 software (Umetrics, Umeå, Sweden). Non-numerical data (values below the LLOQ or above the ULOQ) were treated as missing values. Metabolites with more than 80% of missing values were excluded from multivariate models.

PCA, an unsupervised descriptive tool, was employed to provide an overview of the data. In order to confirm the patterns observed with PCA and to identify pattern-related metabolites, supervised models were constructed to relate metabolic profiles to biological and physiological factors, using OPLS for continuous responses (age and cholesterol) and OPLS-DA for categorical response (gender). The quality and significance of the supervised models was assessed using the predicted variation in Y (Q^2^Y) calculated from 7-fold cross-validation, and the CV-ANOVA *p*-value calculated by Simca software (Analysis of variance of cross-validated predictive residuals). Interpretation of the models was based on S-plots. An S-plot is a scatter plot of metabolites, allowing the influence of each metabolite on the model to be visualized. It consists of plotting the modeled correlation (P_corr_) versus the modeled covariance (p) between the metabolite concentrations and the sample scores on the predictive component. When unit variance scaling is applied to the data matrix to give equal importance to all metabolites whatever their range of concentration (which was the case here), the S-plot is a diagonal line instead of being S-shaped. Metabolites with high absolute P_corr_ values (lower left and upper right corners) are those having the most influence in the response modeling.

## Results

### Biochemical and physiological characteristics of the VARIETE study

Among the 895 recruited subjects, 94 subjects were taking medications and were excluded from the final analysis. Another subject with a very high TBC concentration (12.2 mmol/L) was also excluded. Finally, the HVs group consisted of 800 subjects, whose main characteristics are shown in Table 1. Females represented 383 (47.9%) of the HVs. Age ranged from 18 to 86 years, with a mean of 37.6±17.2. Men had higher BMI, systolic and diastolic blood pressure, blood creatinine and glucose than women. No significant gender difference in TBC was found.

**Table 1:** Main characteristics of the healthy volunteers according to sex.

Subjects	N (M/F)		Mean ± SD	Median	Inter-quartile Range	Extreme values	p^a^
Age (years)	800 (417/383)		37.6±17.2	30		(18;86)	
		M	36.4±16.9				*
		F	38.8±17.5				
18–29	391 (215/176)						
30–39	99 (54/45)						
40–49	94 (46/48)						
50–59	89 (42/47)						
60–69	78 (38/40)						
>70	49 (22/27)						
**Body mass index (kg/m2)**		M	23.5±2.3	23.4	[21.7;25.4]	(19.0;28.1)	***
		F	22.3±2.4	22.0	[20.4;24.0]	(19.0;28.0)	
**Systolic blood pressure (mmHg)**		M	125±10	125	[118;133]	(100;150)	***
		F	117±12	115	[109;124]	(100;150)	
**Diastolic blood pressure (mmHg)**		M	73± 8	72	[69;79]	(50;90)	***
		F	69± 8	69	[63;75]	(50;90)	
**Blood creatinine (μmol/L)**^b^		M	83.3±10.3	82	[76;90]	(52;123)	***
		F	66.7±9.3	66	[60;73]	(39;95)	
**Glycemia (mmol/L)**^c^		M	4.74±0.60	4.70	[4.4;5.1]	(2.8;7.5)	***
		F	4.55±0.56	4.60	[4.2;4.9]	(2.8;6.9)	
**Total blood cholesterol (mmol/L)**^d^		M	4.85±1.02	4.77	[4.17;5.50]	(2.05;7.53)	ns
		F	4.96±1.17	4.85	[4.26;5.70]	(1.06;8.40)	

^a^. Statistical differences between men and women.

*** p<0.001,

*p<0.05

^b^. Conversion factor: 1 μmol/L = 0.113 mg/L

^c^. Conversion factor: 1 mmol/L = 0.180 g/L

^d^. Conversion factor: 1 mmol/L = 0.387 g/L

### Reference plasma metabolite concentrations

The p180 Biocrates kits used in this study allowed the measurement of 185 metabolites in plasma (21 amino acids, 18 biogenic amines, 40 acylcarnitines, 14 lysophosphatidylcholines, 76 phosphatidylcholines, 15 sphingomyelins, and the sum of hexoses). Some metabolites could not be quantified in all subjects, and some others were almost never quantified or detected. Finally, reference values could be obtained for 144 out of the 185 metabolites, corresponding to 21/21 amino acids, 6/18 biogenic amines, 17/40 acylcarnitines, 13/14 lysophosphatidylcholines, 72/76 phosphatidylcholines, 14/15 sphingomyelins and the sum of hexoses. The 21 quantified amino acids are shown in S1 Table. The most abundant amino acid was L-glutamine (0.66 mmol/L), and the least abundant was L-aspartic acid (6.7 μmol/L). Among the 18 biogenic amines analyzed by LC-MS/MS, reference concentrations were established for only 6 of them (N-acetylornithine, asymmetric dimethylarginine, creatinine, kynurenine, serotonin, and taurine). Details on these biogenic amines are shown in S2 Table. Creatinine concentrations measured with the Biocrates kit (74±14 μmol/L) were similar to those obtained by routine biochemistry (75±13 μmol/L). The most abundant acylcarnitine (S3 Table) was free L-carnitine (35±7 μmol/L), followed by acetylcarnitine (6.6±2.3 μmol/L), while most long-chain acylcarnitines were not detected. Reference values for the 13 detected lysophosphatidylcholines and the 72 detected phosphatidylcholines are shown in S4 and S5 Tables, respectively. Nine sphingomyelins and 5 hydroxysphingomyelins were detected (S6 Table). Finally, the mean hexose (sum of hexose, widely represented by glucose: 90–95%) concentration was 5102±595 μmol/L, with a median of 5088 μmol/L (interquartile range 4698–5452) and a range of 3545 to 8898 μmol/L.

### Main sources of metabolome variation in healthy volunteers

Principal components analysis was performed on the dataset containing the concentrations of the detected metabolites for each VARIETE plasma sample. On a PCA score plot, each dot represents a healthy volunteer (or more generally a sample), and dots can be colored using clinical metadata. Interestingly, the dot distribution was very homogeneous. Although HVs were enrolled in 10 French university hospitals in a one year period, cohesion in dots distribution indicated a weak inter-center variation thanks to the numerous efforts made to strictly standardize pre-analytical conditions of plasma collection.

**Fig 1:**
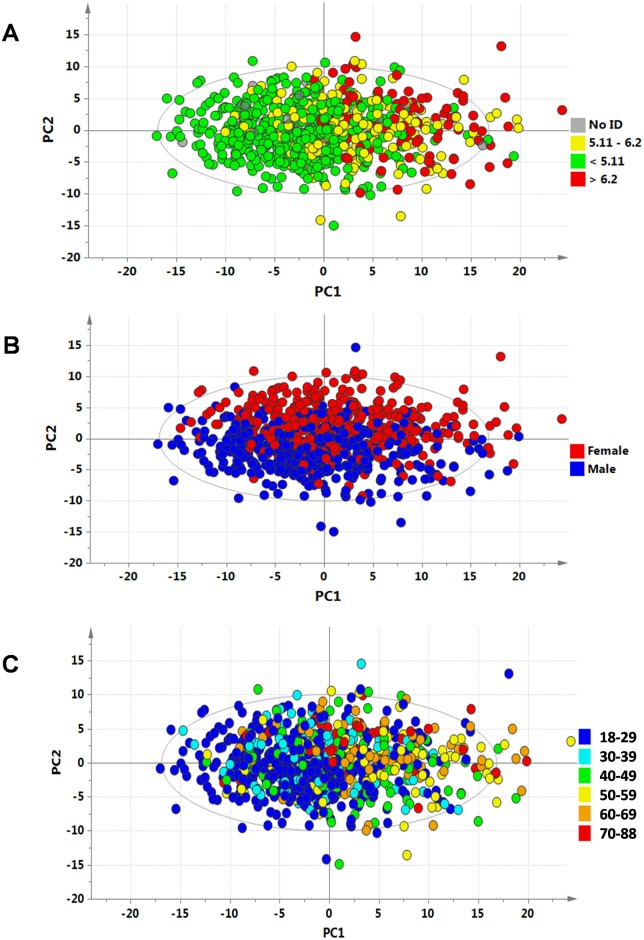
Scores plot from PCA multivariate analyses: PC1 *vs*. PC2 scores plot from PCA of metabolic profiles, colored according to (A) Total Blood Cholesterol, (B) Gender, (C) Age. **(A) Total Blood Cholesterol**. Clinically relevant limits have been set on the TBC concentrations; the red dots (HVs with TBC above 6.2 mmol/L) are on the right, the green dots (HVs with TBC below 5.1 mmol/L) are over-represented on the left, and the yellow dots are in-between. **(B) Gender**. Clouds of red dots (Females) are mainly represented on the upper side of the PC2, while blue dots (Males) are mainly represented on the lower side of the PC2. **(C) Age**. Each age group is represented in different colors, from blue to red; an aging trend is seen along PC1.

The PCA score plot corresponding to the first two principal components showed that PC1 and 2 explained respectively 26% and 9% of the dataset total variance. Possible relationships between the location of the samples in the scores plots and, biological and physiological metadata were investigated visually. Although the dot distribution was very homogenous, some perceptible trends could be observed. The first source of variation in the metabolic profile—held by the PC1 –seemed related to total blood cholesterol (TBC) (Fig 1A). Among 133 HVs with TBC over 6.2 mmol/L, 95% were located on the right side of the PCA (PC1 > 0) and 5% on the left side (PC1 < 0). Coloring the same dots according to male (blue) or female (red) status revealed that the second source of variation in the metabolic profile—held by the PC2 –seemed related to gender (Fig 1B), with 75% of females located in the upper part of the plot (PC2 > 0) and 74% of males located in the lower part of the plot (PC2 < 0). When color was based on age groups, a trend was visualized along the PC1, with HVs over 60 mostly located on the right part of the plot (Fig 1C). This trend was less obvious than the TBC-based pattern though. In order to confirm the patterns observed in PCA and to identify metabolites responsible for these patterns, supervised OPLS models were constructed to relate metabolic profiles to total blood cholesterol, age and gender.

### Impact of total blood cholesterol on the metabolic profile in the whole HVs population

**Fig 2:**
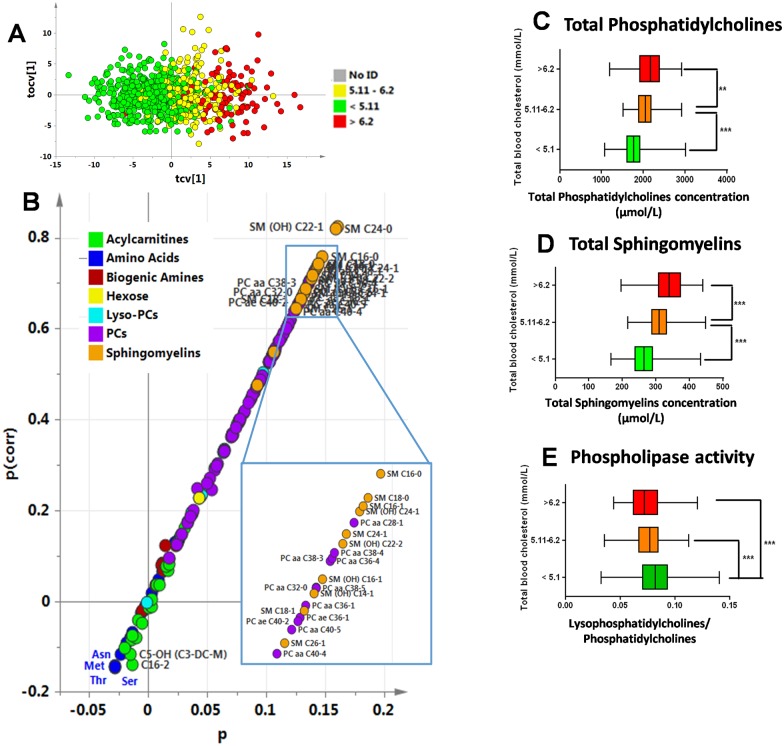
Correlation of TBC with metabolome values in healthy volunteers. **(A) Scores plot from OPLS multivariate analysis, cross-validated score plot resulting from OPLS modeling of Total Blood Cholesterol.** Clinically relevant limits have been set on the TBC concentrations; the red dots represent HVs with TBC above 6.2 mmol/L, the green dots, HVs with TBC below 5.1 mmol/L, and the yellow dots are in-between. **(B) TBC S-plot.** Metabolites in the upper right corner correlate positively with total blood cholesterol. The p axis describes the contribution of each variable to the model. **(C) Total phosphatidylcholines concentration according to total blood cholesterol.** TBC below 5.1 mmol/L (green), between 5.11 and 6.2 mmol/L (orange) and above 6.21 mmol/L (red). **(D) Total sphingomyelins concentration according to total blood cholesterol. (E) Phospholipase activity according to total blood cholesterol.** ** p<0.01;***p<0.001.

OPLS modeling of TBC from metabolic profiles produced a good model (Q^2^Y = 72%, p<0.001). On the OPLS cross-validated scores plot (Fig 2A), subjects with normal cholesterol were mostly located on the left, while subjects with high cholesterol were mostly located on the right. The corresponding S-plot (Fig 2B) showed an unbalanced trend towards positively correlated metabolites with TBC. The metabolites in the upper-right corner of the plot (mostly phosphatidylcholines (PCs) in purple and sphingomyelins (SMs) in gold) correlated with higher TBC concentrations. The correlation level is indicated by the y axis (p_corr_). The box plots (Fig 2C and 2D) summarize PCs and SMs behavior versus clinically relevant TBC thresholds. TBC was below 5.1 mmol/L in 473 HVs, between 5.11 and 6.2 mmol/L in 194 HVs, and above 6.2 mmol/L in 133 HVs. In contrast, phospholipase activity, estimated as the ratio of total lysophosphatidylcholines (LPCs) to total phosphatidylcholines (PCs), was higher in the normal TBC group (<5.1 mmol/L) than in the borderline or high TBC group (p<0.001) (Fig 2E).

### Gender effect on the metabolic profile

**Fig 3:**
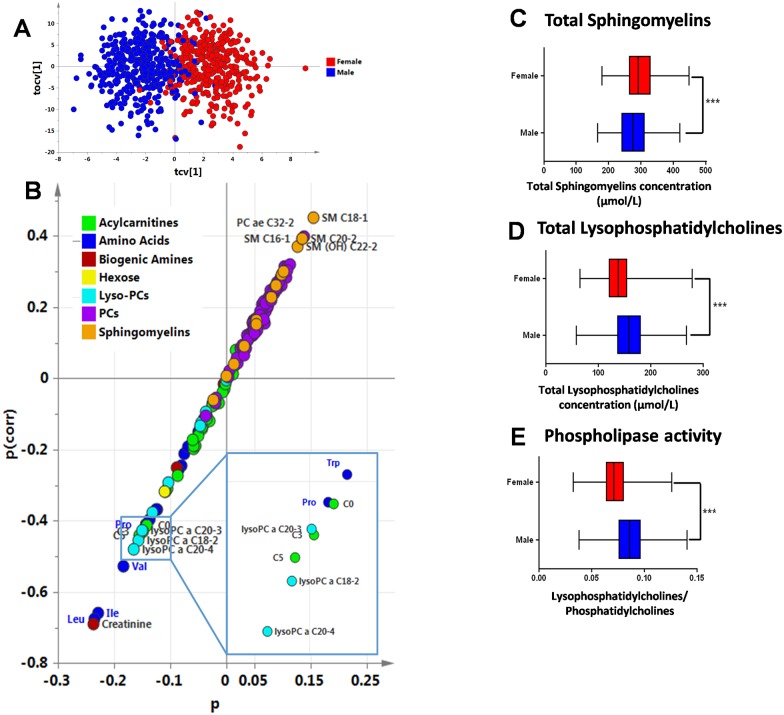
Gender effect on the metabolic profile of healthy volunteers. **(A) Scores plot from OPLS multivariate analysis, cross-validated score plot resulting from OPLS modeling of gender.** Females are represented with red dots, males with blue dots. **(B) Gender S-Plot.** Metabolites in the upper-right corner are higher in women; those in the lower-left corner are higher in males. The p axis describes the contribution of each variable to the model. **(C) Total sphingomyelins concentration according to sex. (D) Total lysophosphatidylcholines concentration according to sex. (E) Phospholipase activity according to sex.** ***p<0.001.

OPLS-DA modeling of gender effect also produced a good model (Q^2^Y = 70%, p<0.001), with males mostly located on the left and females mostly located on the right in the cross-validated scores plot (Fig 3A). The corresponding S-plot (Fig 3B) showed a good balance-between metabolites correlated and anti-correlated with the categorical response (0 = Males; 1 = Females). Creatinine, branched-chain amino acids, small-chain acylcarnitines (C0, C3, C5) and lysophosphatidylcholines had higher concentrations in males (bottom left); while SMs and PCs had higher concentrations in females (top right). Total sphingomyelins concentration was lower in males than in females (p<0.001) (Fig 3C). Total lysophosphatidylcholines concentration was higher in males than in females (p<0.001) (Fig 3D), and so was phospholipase activity (p<0.001) (Fig 3E)

### Effect of age on the metabolic profile

**Fig 4:**
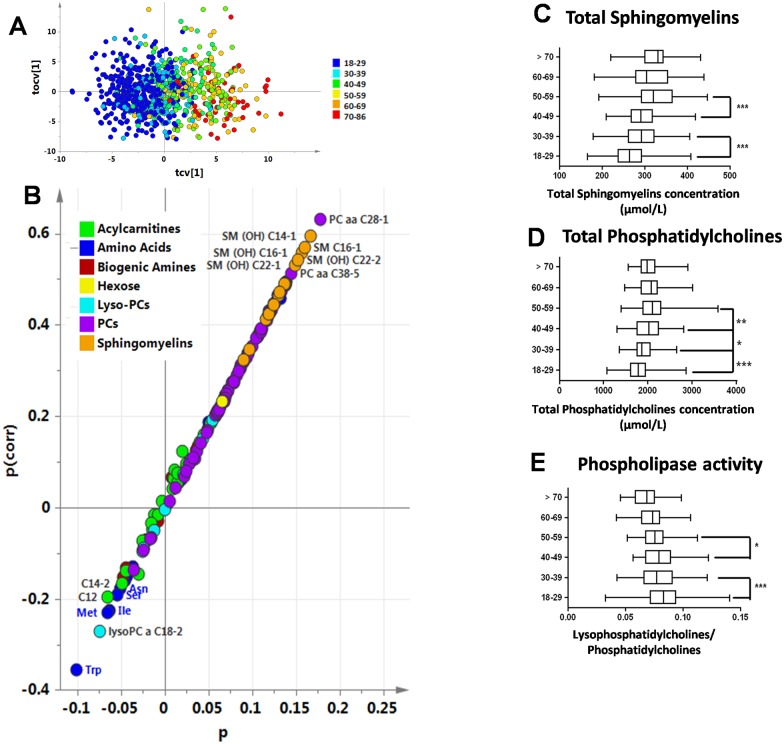
Age effect on the metabolite profile of healthy volunteers. **(A) Scores plot from OPLS multivariate analysis, cross-validated score plot resulting from OPLS modeling of age.** Each age group is represented in different colors, from blue to red. **(B) Age S-plot.** Metabolites in the upper-right corner correlate positively with age, while those in the bottom-left corner correlate negatively with age. The p axis describes the contribution of each variable to the model. (**C) Total sphingomyelins concentration according to the age group. (D) Total phosphatidylcholines concentration according to the age group. (E) Phospholipase activity according to the age group.** *p<0.05; ** p<0.01; ***p<0.001.

A good OPLS model was obtained for age prediction (Q^2^Y = 61%, p<0.001). On the cross-validated scores plot (Fig 4A), subjects under 30 years old were mostly located on the left, while subjects over 60 were mostly located on the right. In the corresponding S-plot (Fig 4B), the top-right corner included the metabolites whose levels increased with age, while the bottom-left corner corresponded to metabolites whose levels fell with age. The gold and purple cluster spots in the upper-right corner represented sphingomyelins and phosphatidylcholines, which increased with age (Fig 4C and 4D respectively). Global phospholipase activity (the ratio of total lysoPCs to total PCs) fell with age (Fig 4E).

## Discussion

The aim of this study was to describe the “reference” physiological range of the human metabolome in a representative sample of 800 French healthy volunteers. Targeted metabolites quantification with a commercial kit allowing comparison with other labs or other patient cohorts had been reported in previous studies based on limited-scale healthy volunteers [2, 5–7]. To our knowledge, the present study of 800 healthy volunteers is one of the largest cohort in which more than 180 small metabolites have been measured.

A valuable strength of this cohort is that participants were well characterized as healthy volunteers, based on their medical history, clinical examination and screening laboratory tests. All HVs had normal routine biochemistry tests, no intercurrent disease, a BMI between 19 and 28 kg/m and they were all in fasting conditions. The recruitment was performed during a relatively short period (around one year) and included subjects aged between 18 and 86. Although 18- to 29-year-old subjects represented around half of the 800 HVs (176 males and 215 females), the study included about 50 male and 50 female HVs in each ten-year age bracket between 30 and 60, and more than 120 healthy volunteers were over 60 (60 males and 67 females). Numerous publications report impact of drugs on biochemical pathways and as a result on blood metabolites concentration. In this context, it is crucial to note that absolutely all healthy volunteers were free of any medication, even elderly participants. Furthermore, HVs were recruited in 10 clinical research units spread over the French territory to ensure a good coverage of the French population. Principal Component Analysis highlighted a good homogeneity of the cohort with no major differences related to the recruitment centers.

To avoid major variations in metabolites measurement, plasma samples were immediately frozen after blood sampling as recommended by Breier et al. [7]. Metabolite concentrations were very homogeneous, testifying to the excellent sample quality, which contributes to the robustness and validity of the results. Moreover, a total of 14 kits were needed to analyze all samples, averaging slight batch effects that could occur. Targeted quantification in human plasma was performed for a large number of metabolites covering several key biochemical pathways, with a commercial kit increasingly used across the community [2, 3, 6–8, 10, 11] and able to provide comparable results from one lab to another. Thereby, contrarily to several other metabolomic results, specific to the lab that performed the study, ranges of variation obtained for healthy volunteers in the present study can be used to compare results from other cohorts of patients, even originating from another lab. Moreover, Brier et al. [7] and more recently Carayol et al. [12] evaluated within-person variability and showed that most metabolite concentrations were highly stable over time in a fasted individual and that a single time point measurement is assumed to be sufficient for a targeted metabolomic analysis of most metabolites.

Metabolomics gives an account of the integrative and dynamic interactions of small biochemical molecules. In biological systems, the set of biochemical molecules is in perpetual movement, to act as the building blocks for larger biochemical entities, to regulate biochemical processes, and to support metabolic reactions sustaining life [1]. While classical biochemistry tests give an account of a pathology or an organ dysfunction through the combination of a restricted number of molecules levels that merely rely upon the descriptions of sets of symptom, metabolomics is a “without a priori method”, that allows a direct readout of biological processes, bringing light on the complexity of intertwined small molecules pathways. Metabolomic approaches associated with statistical analyses are nowadays able to define a metabolic profile associated with pathology, a disease risk prediction, a drug intake and adverse drug reactions. For the latter applications, a new field of personalized and stratified medicine is emerging called pharmacometabonomics [13, 14].

The targeted approach used in the present study gives an overview of four main metabolic areas: amino acids (and biogenic amines), glucose (sum of hexose which is widely represented by glucose (90–95%)), fatty acids (40 acylcarnitines) and lipid metabolism (glycerophospholipids and sphingolipids). Although the entire metabolome is not covered by this method, biochemical pathways involved in several pathologies can be studied. For example, in neurology, amino acids and biogenic amines allow exploring neurotransmitter function (glutamate, dopamine, serotonin) or urea cycle dysregulation; acylcarnitines may shed light on neurodegeneration associated with mitochondrial metabolism impairment; lipid metabolism enables exploring myelin sheaths, which main components are sphingomyelins. In diabetes, amino acids and biogenic amines can point out the involvement of branched-chain amino acids in insulin resistance or the relationship of tryptophan and kynurenine with immune / inflammatory processes; acylcarnitines can help to assess mitochondrial function which affects inflammation and lipid accumulation in the liver; lipid metabolism may provide information on low grade inflammation, as lipids are considered as inflammatory mediators; the sum of hexoses can point out disturbed glucose homeostasis.

In this study, total blood cholesterol was one of the main sources of variation. TBC concentrations have been found to be significantly associated with 94 metabolites in two independent cohorts, namely the German KORA cohort (3044 subjects from independent population-based epidemiological surveys and from follow-up studies of participants living in the region of Augsburg, Southern Germany) and the TwinUK cohort (a British registry composed of 1176 adult twins recruited from the UK general population) [4]. Metabolite measurements in these two cohorts were made with the Biocrates AbsoluteIDQ p150 kit. Most of the 94 metabolites that were significantly associated with TBC in both KORA and TwinUK samples also showed significant associations with TBC in the present study (mainly phosphatidylcholines and sphingomyelins). Of note, phosphatidylcholines (and lysophosphatidylcholines) represent by mass more than 76% of total glycerophospholipids in healthy volunteers [15]. Although phosphatidylcholines had been described as major phospholipids in lipoproteins and cellular membranes for a long time [16], several recent advances have expanded on the notion that PCs are not just a kind of cellular bricks [17]. Such new roles include gene regulation: PC 16:0/18:1 have been described as an endogenous ligand for the nuclear receptor PPAR alpha in hepatocytes [18], a transcription factor regulating the expression of many genes that govern lipid metabolism [19]. There is also evidence for at least two distinct roles for PCs in insulin transduction, according to the number of double bonds on fatty acyl chains [20, 21]. On one hand, Ersoy et al. [20] showed a phosphatidylcholine-dependent mechanism that suppresses insulin signaling downstream of its receptor in a human cell line. This indirect mechanism is induced by PC-Transfer protein, which preferentially binds to PCs with more unsaturated sn-2 fatty acyl chains [22–24]. On the other hand, Sakai et al. [21] showed that saturated PCs may be indirectly involved in regulating glucose homeostasis by providing substrate for the diacylglycerol kinase delta which regulates glucose uptake in muscle cells in mouse models. Although phosphatidylcholines measured by the Biocrates kit are identified by their total acyl/alkyl chain content as opposed to their precise chemical structure, in the present study, most of increasing PCs associated with high TBC contained 2 or more double bonds on fatty acyl chains (PC aa C38-4; PC aa C38-3; PC aa C36-4; PC aa C38-5; PC ae C40-2; PC aa C40-5; PC aa C40-4). These unsaturated PCs could activate PC-Transfer Protein complex formation, involved in decreasing insulin signaling, indicating a potential mechanistic role of PCs in the development of insulin resistance associated with high concentration of total blood cholesterol in plasma.

Gender was also an important determinant of the human plasma metabolome in this study, consistent with previous reports [10, 25, 26]. Some metabolites are known to be higher in males than in females, such as creatinine [27], branched-chain amino acids [28], short-chain acylcarnitines (C0, C3, C5) [29] and lysophosphatidylcholines [25]. We found that SMs were higher in females than in males, in accordance with Ishikawa’s study of 60 Caucasian subjects [30] and Krumsiek’s study of the German KORA cohort [26]. Although a previous study suggests that estrogens may be involved in the regulation of SMs metabolism [31], Nikkila et al. showed that higher levels of sphingomyelins were detected in females during childhood, from birth to 4 years old, before any influence of sexual hormones [32]. Moreover, Ishikawa et al. [30] showed a comparable SMs level in young and elderly women (55–64 years old), despite a markedly decreased estrogen level in postmenopausal elderly women. Circulating SMs levels have been associated with many common complex chronic diseases including cardiomyopathy, insulin resistance, coronary heart disease and type 2 diabetes [33, 34]. Results of our analysis shed light on sexual dimorphism of the human metabolome and provide clues on biochemical mechanisms that might explain observed differences in susceptibility, time course of the development of common diseases or response to drugs between males and females.

Age-related changes in the concentrations of various metabolites have also been observed in several other studies [35, 36]. Matsumoto et al. found that elevated LPCs concentrations were positively associated with atherosclerosis, ischemia and diabetes [37]. These cardiovascular risks are also well known to be positively associated with increased TBC and advanced age. In the large VARIETE study, we didn’t find any correlation between total LPCs and age. The phosphatidylcholines reference values of the present study were slightly lower than those reported in 100 HVs from the EPIC-Potsdam cohort [6], probably because the 100 HVs recruited by Floegel et al. were aged between 35 and 65, compared to the present 18–86 year-old 800 HVs, 391 of whom were aged between 18 and 29. Interestingly, a positive correlation was found between age and sphingomyelins and total phosphatidylcholines concentrations, two classes of molecules with essential roles in cell membrane integrity and function [38]. Aging is a complex, continuous and dynamic process including alteration of cell membrane composition, mitochondrial metabolism and low-grade systemic inflammation connected to oxidative stress [35]. It is well known that oxidative stress has dramatic influences on biological membranes through the peroxidation of lipids, which in turn results in altered membrane fluidity and altered function of membrane embedded proteins [39]. Sphingomyelins and their derivatives play important roles for the regional organization of transmembrane and membrane associated protein thereby modulating intracellular transport, signal transduction (production of diacylglycerol, a second messenger) and metabolism [40]. Sphingomyelinase (SMase) is an enzyme responsible for SMs degradation to ceramides and oxidative stress can accelerate SMs degradation [41, 42]. Cells able to adapt to chronic oxidative stress alter SMs metabolism by reducing SMase activity and making major changes in membrane composition, leading to stabilization [43]. Low levels of SMs have been associated to neurodegenerative diseases [44], atherosclerosis [45], and cardiovascular disease [33]. Although SMase activity has been reported as increasing with age [46], a positive correlation between SMs and age was found in the present study. Other studies reported a similar positive association [35, 36], and Gonzalez-Covarrubias et al. [47] and Vaarhost et al. [48] associated higher levels of sphingomyelins to longevity and healthy aging. Healthy aging individuals could have effective mechanisms to protect cells from oxidative stress, modifying SMs metabolism, reducing SMs degradation, which would indicate a potential positive role of SMs in response to oxidative stress, in order to maintain cell membrane composition and function. Contrarily to total LPCs for which no association with age was found, total PCs increased with age, particularly unsaturated PCs. OPLS analyses showed that 24 PCs with two or more unsaturations on their fatty acyl chains and 11 PCs with at most one double bond were positively associated with age (PC ae C40:2; PC aa C38:5; PC aa C38:3; PC aa C40:6; PC aa C36:6; PC aa C34:4; PC ae C38:3; PC aa C40:5; PC aa C32:3; PC ae C32:2; PC ae C30:2; PC ae C38:6; PC aa C38:6; PC aa C36:5; PC aa C38:4; PC aa C36:4; PC ae C40:6; PC aa C34:3; PC ae C36:2; PC aa C32:2; PC ae C36:5; PC ae C38:5; PC aa C34:2). As mentioned for high TBC, increased unsaturated PCs plasma level in elderly HVs could be related to a decrease in insulin signaling, as aging is related to a decrease in insulin sensitivity.

The TBC-related and age-related metabolic profiles exhibited some similarities, with most of the quantified sphingomyelins and many phosphatidylcholines being related to both age and TBC. This was expected, as aging was associated with increased cholesterol levels in our cohort. Nevertheless, the TBC-related and age-related metabolic profiles were not identical. For example, the OPLS analyses showed that citrulline levels increased and tryptophan levels decreased with age, but neither metabolite correlated with TBC, while increased lyso-PC C18:0 with higher TBC was observed without any association with age. The association between the metabolic profiles and TBC was further confirmed in the subjects aged between 26 and 40 (in whom there was no correlation between cholesterol and age), and also by comparing subjects with high cholesterol concentrations with matched subjects who had desirable cholesterol concentrations and similar values for the other clinical metadata (gender, menopausal status, age, BMI, glycemia, and blood pressure). The TBC-related metabolic profiles obtained with these two approaches were very similar to those obtained in the whole cohort (the same sphingomyelins and phosphatidylcholines were associated with higher TBC concentrations in the three models).

Our study has limitations, especially because other factors that can induce variation of metabolism such as genetic inheritance [49], diet [50] and exercise [5], were not studied. In order to reduce genetic variation, all healthy volunteers recruited in VARIETE study were Caucasian [51]. Concerning the impact of diet effect on the metabolome, we tried to minimize the dietary influence by collecting plasma samples on overnight-fasted subjects, as performed for classical biochemistry exploration. In addition, the fasting state of all the HVs makes possible the comparison of metabolic profiles obtained from cohorts of patients with various diseases, equally in fasting conditions, with these reference values. Nevertheless, it cannot be ruled out that blood composition can be affected by food-related metabolites but this reflects physiological variability in the general population. Considering our healthy volunteers came from 10 different regions, the probability of a bias due to a dietary component shared among all participants is weak. In addition, even if a dietary component was consumed by all our healthy volunteers, the probability to detect an association between this component and one metabolite is still very low [52]. Physical exercise can also be another source of variation in metabolism. However, it is unlikely that drastic changes in diet or exercise habits which could have occurred in a few subjects would have change the main messages obtained from the rest of the population. Overall, taking into account the very large number and diversity of subjects in the cohort and the fact that they were recruited in 10 centers spread over the French territory, the cohort is likely to be representative of the normal healthy French metabolome in terms of diet and exercise habits. Nevertheless, lack of these two clinical parameters is a limitation of our study and reference values obtained in other populations with very different dietary and exercise habits could provide evidence for proposing different reference values from those given by our study.

Our findings contribute to the understanding of the human metabolism. The reference physiological values of the plasma metabolome obtained in this large healthy French population will be a valuable tool for interpreting values obtained in various pathological situations. The present work highlights that some metabolites are strongly influenced by cholesterol level, gender or age and special care in study designs should be taken when investigating variations of these metabolites in metabolomics studies.

## Supporting information

S1 Table Reference values for 21 amino acids. (DOCX)

S2 Table Reference values for 18 biogenic amines. (DOCX)

S3 Table Reference values for 40 acylcarnitines. (DOCX)

S4 Table Reference values for 14 lysophosphatidylcholines. (DOCX)

S5 Table Reference values for 76 Phosphatidylcholines. (DOCX)

S6 Table Reference values for 5 hydroxysphingomyelins and 10 sphingomyelins. (DOCX)

## Abbreviations

FIA: flow injection analysis
HVs: healthy volunteers
LC: liquid chromatography
LLOQ: lower limit of quantification
LOD: limit of detection
LPCs: lysophosphatidylcholines
MS/MS: tandem mass spectrometry
ND: not detected
OPLS: orthogonal projection to latent structures
OPLS-DA: OPLS-discriminant analysis
PCA: principal components analysis
PCs: phosphatidylcholines
SMs: sphingomyelins
TBC: total blood cholesterol
ULOQ: upper limit of quantification
