# Supplementary material for: The human plasma-metabolome: Reference values in 800 French healthy volunteers; impact of cholesterol, gender and age
Source: PLoS One. 2017 Mar 9;12(3):e0173615. doi: 10.1371/journal.pone.0173615 (PMC5344496; doi:10.1371/journal.pone.0173615)
Supplement: S1 Table — (DOCX) [file pone.0173615.s001.docx]

**S1 Table: Reference values for 21 Amino acids**

|  |  |  | **Mean ± SD (µmol/L)** | **Median** | **Inter-quartile Range** | **Extreme values** | **LLOQ** | **% ND** |
| --- | --- | --- | --- | --- | --- | --- | --- | --- |
|  |  |  |  |  |  |  |  |  |
|  | L-Alanine |  | 319.4±71.7 | 315.3 | [266.0;365.0] | (114.2;608.7) | 20.0 | 0 |
|  | L-Arginine |  | 81.4±19.3 | 80.7 | [68.5;93.4] | (16.2;147.6) | 5.00 | 0 |
|  | L-Asparagine |  | 48.4±9.29 | 47.5 | [42.1;53.4] | (26.8;92.0) | 5.00 | 0 |
|  | L-Aspartic acid |  | 6.26±2.34 | 6.10 | [ND;7.53] | (ND;20.1) | 5.00 | 28 |
|  | L-Citrulline |  | 29.8±7.91 | 28.8 | [24.4;34.3] | (8.66;63.6) | 5.00 | 0 |
|  | L-Glutamine |  | 657.9±106.2 | 652.9 | [582.1;722.2] | (355.6;1069.1) | 20.0 | 0 |
|  | L-Glutamic acid | | 46.2±21.4 | 42.2 | [30.8;58.7] | (9.12;192.3) | 10.0 | 0 |
|  | Glycine |  | 255.4±65.9 | 244.6 | [211.7;289.0] | (105.5;549.0) | 25.0 | 0 |
|  | L-Histidine |  | 89.3±10.8 | 89.4 | [82.3;95.6] | (59.3;154.8) | 5.00 | 0 |
|  | L-Isoleucine |  | 77.5±15.4 | 76.5 | [66.0;88.0] | (41.0;144.0) | 5.00 | 0 |
|  | L-Leucine |  | 150.1±27.7 | 147.6 | [129.3;171.2] | (85.0;241.9) | 10.0 | 0 |
|  | L-Lysine |  | 197.4±30.7 | 195.9 | [178.4;215.1] | (107.2;302.5) | 10.0 | 0 |
|  | L-Methionine |  | 25.4±5.05 | 24.9 | [22.0;28.3] | (12.8;48.0) | 5.00 | 0 |
|  | L-Ornithine |  | 69.0±18.0 | 66.1 | [56.5;79.7] | (28.2;144.2) | 5.00 | 0 |
|  | L-Phenylalanine |  | 60.0±7.75 | 59.4 | [54.6;64.5] | (37.4;102.9) | 5.00 | 0 |
|  | L-Proline |  | 188.3±55.0 | 179.4 | [146.9;220.3] | (81.4;525.5) | 10.0 | 0 |
|  | L-Serine |  | 115.1±25.0 | 112.6 | [99.4;129.8] | (53.4;262.6) | 5.00 | 0 |
|  | L-Threonine |  | 127.9±28.2 | 124.5 | [109.3;144.0] | (51.9;234.4) | 5.00 | 0 |
|  | L-Tryptophan |  | 63.0±10.7 | 62.7 | [55.8;69.6] | (35.9;105.2) | 5.00 | 0 |
|  | L-Tyrosine |  | 62.9±12.2 | 61.4 | [54.1;70.9] | (30.8;125.0) | 5.00 | 0 |
|  | L-Valine |  | 243.6±45.2 | 241.1 | [210.5;271.6] | (131.2;422.6) | 10.0 | 0 |
|  |  |  |  |  |  |  |  |  |

LLOQ: Lower limit of quantification, ND: Not detected (below LLOQ)
