# Supplementary material for: The human plasma-metabolome: Reference values in 800 French healthy volunteers; impact of cholesterol, gender and age
Source: PLoS One. 2017 Mar 9;12(3):e0173615. doi: 10.1371/journal.pone.0173615 (PMC5344496; doi:10.1371/journal.pone.0173615)
Supplement: S2 Table — (DOCX) [file pone.0173615.s002.docx]

**S2 Table: Reference values for 18 biogenic amines**

|  |  | Mean ± SD **(µmol/L)** | Median | Inter-quartile Range | Extreme values | LLOQ | % ND |
| --- | --- | --- | --- | --- | --- | --- | --- |
|  |  |  |  |  |  |  |  |
|  | N-Acetylornithine | 1.03±0.78 | 0.88 | [0.55;1.32] | (ND;11.52) | 0.50 | 21 |
|  | Asymmetric dimethylarginine | 0.45±0.11 | 0.44 | [0.38;0.51] | (ND;0.86) | 0.25 | 1 |
|  | alpha-Aminoadipic acid | ND | ND | ND | (ND;2.62) | 1.00 | 78 |
|  | Carnosine | ND | ND | ND | ND | 0.50 | 100 |
|  | Creatinine | 74.1±13.6 | 73.8 | [64.1;83.0] | (40.0;124.9) | 10.0 | 0 |
|  | Histamine | ND | ND | ND | ND | 1.00 | 100 |
|  | Kynurenine | 2.21±0.57 | 2.14 | [1.79;2.54] | (ND;4.86) | 1.00 | <1 |
|  | Methionine Sulfoxide | ND | ND | ND | (ND;2.37) | 1.00 | 99 |
|  | Nitrotyrosine | ND | ND | ND | ND | 1.00 | 100 |
|  | cis-4-Hydroxy-L-proline | ND | ND | ND | ND | 1.00 | 100 |
|  | Phenylethylamine | ND | ND | ND | ND | 0.10 | 100 |
|  | Putrescine | ND | ND | ND | (ND;0.27) | 0.10 | 45 |
|  | Symmetric dimethylarginine | ND | ND | ND | ND | 1.00 | 100 |
|  | Serotonin | 0.41±0.33 | 0.33 | [0.17;0.57] | (ND;2.77) | 0.10 | 15 |
|  | Spermidine | ND | ND | ND | (ND;0.58) | 0.25 | 54 |
|  | Spermine | ND | ND | ND | (ND;0.74) | 0.50 | >99 |
|  | Taurine | 93.0±35.7 | 87.0 | [68.2;111.6] | (28.4;>200) | 2.50 | 0 |
|  | Total dimethylarginine | ND | ND | ND | (ND;2.01) | 1.25 | 97 |

LLOQ: Lower limit of quantification, ND: Not detected (below LLOQ)
