# Supplementary material for: The human plasma-metabolome: Reference values in 800 French healthy volunteers; impact of cholesterol, gender and age
Source: PLoS One. 2017 Mar 9;12(3):e0173615. doi: 10.1371/journal.pone.0173615 (PMC5344496; doi:10.1371/journal.pone.0173615)
Supplement: S3 Table — (DOCX) [file pone.0173615.s003.docx]

**S3 Table: Reference values for 40 acylcarnitines**

|  |  | Mean ± SD **(µmol/L)** | Median | Inter-quartile Range | Extreme values | LOD | % ND |
| --- | --- | --- | --- | --- | --- | --- | --- |
|  |  |  |  |  |  |  |  |
| **Sum of acylcarnitine (µmol/L)** | | 45.7±8.0 | 45.6 | [40.1;50.5] | (25.6;79.8) |  |  |
|  |  |  |  |  |  |  |  |
|  | L-Carnitine | 35.3±7.0 | 35.2 | [30.3;39.7] | (19.0;67.1) | 2.20 | 0 |
|  | Decanoylcarnitine | 0.31±0.13 | 0.28 | [0.22;0.37] | (ND;1.20) | 0.13 | 31 |
|  | Decenoylcarnitine | ND | ND | ND | (ND;0.47) | 0.12 | 88 |
|  | Decadienylcarnitine | ND | ND | ND | (ND;0.54) | 0.07 | 98 |
|  | Dodecanoylcarnitine | ND | ND | ND | (ND;0.40) | 0.07 | 50 |
|  | Dodecanedioylcarnitine | ND | ND | ND | ND | 0.10 | 100 |
|  | Dodecenoylcarnitine | ND | ND | ND | (ND;0.42) | 0.11 | 81 |
|  | Tetradecanoylcarnitine | ND | ND | ND | (ND;0.12) | 0.04 | 54 |
|  | Tetradecenoylcarnitine | 0.13±0.04 | 0.12 | [0.10 ;0.15] | (0.05;0.46) | 0.01 | 0 |
|  | Hydroxytetradecenoylcarnitine | ND | ND | ND | (ND;0.06) | 0.01 | 59 |
|  | Tetradecadienylcarnitine | 0.04±0.02 | 0.03 | [0.03;0.04] | (ND;0.16) | 0.01 | 14 |
|  | Hydroxytetradecadienylcarnitine | ND | ND | ND | (ND;0.03) | 0.01 | 78 |
|  | Hexadecanoylcarnitine | 0.11±0.03 | 0.10 | [0.09;0.12] | (0.04;0.23) | 0.01 | 0 |
|  | Hydroxyhexadecanoylcarnitine | ND | ND | ND | (ND ;0.028) | 0.007 | 89 |
|  | Hexadecenoylcarnitine | ND | ND | ND | (ND;0.11) | 0.04 | 86 |
|  | Hydroxyhexadecenoylcarnitine | ND | ND | ND | (ND;0.03) | 0.008 | 73 |
|  | Hexadecadienylcarnitine | ND | ND | ND | (ND;0.05) | 0.01 | 78 |
|  | Hydroxyhexadecadienylcarnitine | ND | ND | ND | ND | 0.02 | 100 |
|  | Octadecanoylcarnitine | 0.04±0.01 | 0.04 | [0.04;0.05] | (ND;0.08) | 0.01 | <1 |
|  | Octadecenoylcarnitine | 0.12±0.03 | 0.11 | [0.09;0.13] | (0.03;0.32) | 0.02 | 0 |
|  | Hydroxyoctadecenoylcarnitine | ND | ND | ND | ND | 0.02 | 100 |
|  | Octadecadienylcarnitine | 0.05±0.02 | 0.05 | [0.04;0.06] | (ND;0.18) | 0.01 | <1 |
|  | Acetylcarnitine | 6.56±2.26 | 6.13 | [4.98;7.69] | (2.21;19.31) | 0.08 | 0 |
|  | Propionylcarnitine | 0.34±0.10 | 0.32 | [0.27;0.39] | (0.14;1.05) | 0.04 | 0 |
|  | Hydroxybutyrylcarnitine | 0.04±0.02 | 0.03 | [0.03;0.04] | (ND;0.19) | 0.02 | 39 |
|  | Hydroxypropionylcarnitine | ND | ND | ND | ND | 0.06 | 100 |
|  | Propenoylcarnitine | ND | ND | ND | (ND;0.09) | 0.02 | 98 |
|  | Butyrylcarnitine | 0.21±0.09 | 0.19 | [0.16;0.24] | (0.09;0.89) | 0.04 | 0 |
|  | Butenylcarnitine | ND | ND | ND | (ND;0.19) | 0.02 | 90 |
|  | Valerylcarnitine | 0.14±0.04 | 0.14 | [0.11;0.17] | (ND;0.29) | 0.03 | 2 |
|  | Glutarylcarnitine (Hydroxyhexanoylcarnitine) | ND | ND | ND | (ND;0.05) | 0.01 | 72 |
|  | Methylglutarylcarnitine | ND | ND | ND | (ND;0.19) | 0.05 | 91 |
|  | Hydroxyvalerylcarnitine (Methylmalonylcarnitine) | ND | ND | ND | (ND;0.44) | 0.07 | 77 |
|  | Tiglylcarnitine | ND | ND | ND | (ND;0.09) | 0.04 | 99 |
|  | Glutaconylcarnitine | ND | ND | ND | (ND;0.06) | 0.01 | 92 |
|  | Hexanoylcarnitine (Fumarylcarnitine) | 0.06±0.02 | 0.06 | [0.04;0.07] | (ND;0.24) | 0.03 | 41 |
|  | Hexenoylcarnitine | ND | ND | ND | (ND;0.08) | 0.02 | 92 |
|  | Pimelylcarnitine | 0.03±0.02 | 0.03 | [0.02;0.04] | (ND;0.17) | 0.02 | 44 |
|  | Octanoylcarnitine | 0.18±0.07 | 0.16 | [0.13;0.22] | (ND;0.77) | 0.11 | 41 |
|  | Nonaylcarnitine | 0.08±0.03 | 0.07 | [0.06;0.09] | (ND;0.20) | 0.02 | 22 |

LOD: Limit of detection, ND: Not detected (below LOD)
