# Supplementary material for: The human plasma-metabolome: Reference values in 800 French healthy volunteers; impact of cholesterol, gender and age
Source: PLoS One. 2017 Mar 9;12(3):e0173615. doi: 10.1371/journal.pone.0173615 (PMC5344496; doi:10.1371/journal.pone.0173615)
Supplement: S4 Table — (DOCX) [file pone.0173615.s004.docx]

**S4 Table: Reference values for 14 lysophosphatidylcholines**

|  |  | Mean ± SD **(µmol/L)** | Median | Inter-quartile Range | Extreme values | LOD | % ND |
| --- | --- | --- | --- | --- | --- | --- | --- |
|  |  |  |  |  |  |  |  |
| **Sum of lysophosphatidylcholines (µmol/L)** | | 155.9±32.0 | 152.0 | [134.9;175.2] | (63.2;284.7) |  |  |
|  |  |  |  |  |  |  |  |
|  | lysoPC a C14:0 | ND | ND | ND | ND | 4.50 | 100 |
|  | lysoPC a C16:0 | 73.4±12.9 | 71.7 | [63.0;81.9] | (31.5;133.7) | 0.06 | 0 |
|  | lysoPC a C16:1 | 2.16±0.64 | 2.04 | [1.75;2.48] | (0.70;6.32) | 0.05 | 0 |
|  | lysoPC a C17:0 | 1.64±0.40 | 1.60 | [1.32;1.88] | (0.57;3.06) | 0.02 | 0 |
|  | lysoPC a C18:0 | 21.8±4.97 | 21.4 | [18.4;24.9] | (6.74;41.74) | 0.16 | 0 |
|  | lysoPC a C18:1 | 17.5±4.62 | 16.9 | [14.4;20.2] | (5.79;37.67) | 0.05 | 0 |
|  | lysoPC a C18:2 | 26.6±9.20 | 25.3 | [19.8;31.3] | (9.12;74.2) | 0.05 | 0 |
|  | lysoPC a C20:3 | 1.75±0.56 | 1.65 | [1.36;2.07] | (0.45;3.97) | 0.07 | 0 |
|  | lysoPC a C20:4 | 5.14±1.50 | 4.99 | [4.07;5.93] | (1.85;12.2) | 0.03 | 0 |
|  | lysoPC a C24:0 | 0.16±0.04 | 0.16 | [0.13;0.19] | (ND;0.34) | 0.05 | 7 |
|  | lysoPC a C26:0 | 0.23±0.07 | 0.21 | [0.18;0.26] | (ND;0.55) | 0.04 | 4 |
|  | lysoPC a C26:1 | 0.11±0.04 | 0.10 | [0.08;0.13] | (0.04;0.34) | 0.01 | 0 |
|  | lysoPC a C28:0 | 0.23±0.07 | 0.22 | [0.19;0.27] | (ND;0.56) | 0.06 | 3 |
|  | lysoPC a C28:1 | 0.36±0.11 | 0.34 | [0.28;0.42] | (0.14;0.79) | 0.01 | 0 |

LOD: Limit of detection, ND: Not detected (below LOD)
