# Supplementary material for: The human plasma-metabolome: Reference values in 800 French healthy volunteers; impact of cholesterol, gender and age
Source: PLoS One. 2017 Mar 9;12(3):e0173615. doi: 10.1371/journal.pone.0173615 (PMC5344496; doi:10.1371/journal.pone.0173615)
Supplement: S5 Table — (DOCX) [file pone.0173615.s005.docx]

**S5 Table: Reference values for 76 Phosphatidylcholines**

|  |  | Mean ± SD **(µmol/L)** | Median | Inter-quartile Range | Extreme values | LOD | % ND |
| --- | --- | --- | --- | --- | --- | --- | --- |
|  |  |  |  |  |  |  |  |
| **Sum of Phosphatidylcholines (µmol/L)** | | 1908±307 | 1875 | [1702;2098] | (1077;3008) |  |  |
|  |  |  |  |  |  |  |  |
|  | PC aa C24:0 | 0.10±0.04 | 0.10 | [0.07;0.11] | (ND;0.28) | 0.05 | 30 |
|  | PC aa C26:0 | ND | ND | ND | (ND;1.3) | 1.1 | >99 |
|  | PC aa C28:1 | 3.04±0.81 | 2.91 | [2.45;3.53] | (1.32;7.69) | 0.02 | 0 |
|  | PC aa C30:0 | 4.73±1.65 | 4.44 | [3.59;5.46] | (1.74;12.71) | 0.13 | 0 |
|  | PC aa C30:2 | ND | ND | ND | ND | 0.01 | 100 |
|  | PC aa C32:0 | 15.1±3.01 | 14.8 | [13.0;17.1] | (8.01;25.9) | 0.02 | 0 |
|  | PC aa C32:1 | 16.6±7.90 | 14.4 | [11.4;19.7] | (4.42;64.6) | 0.003 | 0 |
|  | PC aa C32:2 | 4.02±1.47 | 3.84 | [2.99;4.80] | (1.03;11.76) | 0.003 | 0 |
|  | PC aa C32:3 | 0.49±0.12 | 0.47 | [0.40;0.56] | (0.21;1.03) | 0.002 | 0 |
|  | PC aa C34:1 | 242.2±51.3 | 234.5 | [208.0;271.9] | (126.1;490.0) | 0.05 | 0 |
|  | PC aa C34:2 | 413.3±69.2 | 407.6 | [366.0;456.5] | (218.0;670.7) | 0.04 | 0 |
|  | PC aa C34:3 | 15.9±4.61 | 15.1 | [12.8;18.5] | (6.28;37.2) | 0.006 | 0 |
|  | PC aa C34:4 | 1.87±0.66 | 1.77 | [1.37;2.25] | (0.55;4.82) | 0.005 | 0 |
|  | PC aa C36:0 | 2.52±0.89 | 2.42 | [1.93;3.04] | (0.42;7.49) | 0.22 | 0 |
|  | PC aa C36:1 | 57.1±13.5 | 55.3 | [48.0;64.3] | (27.7;119.8) | 0.02 | 0 |
|  | PC aa C36:2 | 250.7±47.2 | 245.5 | [220.4;280.2] | (113.2;428.0) | 0.02 | 0 |
|  | PC aa C36:3 | 129.9±26.4 | 127.2 | [112.7;145.5] | (66.0;245.3) | 0.02 | 0 |
|  | PC aa C36:4 | 189.4±38.8 | 184.3 | [162.7;212.9] | (92.0;349.6) | 0.02 | 0 |
|  | PC aa C36:5 | 29.3±15.5 | 25.8 | [19.3;34.7] | (8.70;142.2) | 0.01 | 0 |
|  | PC aa C36:6 | 1.16±0.47 | 1.10 | [0.84;1.40] | (0.38;5.70) | 0.001 | 0 |
|  | PC aa C38:0 | 3.48±0.95 | 3.36 | [2.83;3.98] | (1.41;7.30) | 0.02 | 0 |
|  | PC aa C38:1 | 1.23±0.46 | 1.17 | [0.91;1.47] | (0.05;3.97) | 0.007 | 0 |
|  | PC aa C38:3 | 47.3±11.3 | 45.2 | [39.4;52.9] | (22.0;98.5) | 0.02 | 0 |
|  | PC aa C38:4 | 101.6±22.6 | 99.1 | [85.7;115.7] | (45.1;195.7) | 0.01 | 0 |
|  | PC aa C38:5 | 54.2±13.2 | 52.2 | [44.9;61.6] | (24.4;110.1) | 0.006 | 0 |
|  | PC aa C38:6 | 88.0±25.0 | 86.5 | [70.5;103.0] | (27.5;214.2) | 0.001 | 0 |
|  | PC aa C40:1 | 0.39±0.10 | 0.39 | [0.34;0.45] | (ND;0.93) | 0.25 | 22 |
|  | PC aa C40:2 | 0.32±0.12 | 0.29 | [0.25;0.36] | (0.14;2.07) | 0.009 | 0 |
|  | PC aa C40:3 | 0.55±0.18 | 0.52 | [0.44;0.61] | (0.26;3.68) | 0.001 | 0 |
|  | PC aa C40:4 | 3.15±0.74 | 3.05 | [2.62;3.54] | (1.38;6.66) | 0.006 | 0 |
|  | PC aa C40:5 | 9.40±2.50 | 8.99 | [7.67;10.84] | (4.10;23.16) | 0.001 | 0 |
|  | PC aa C40:6 | 27.8±8.8 | 26.5 | [21.9;33.2] | (8.36;67.5) | 0.10 | 0 |
|  | PC aa C42:0 | 0.65±0.18 | 0.63 | [0.53;0.75] | (0.26;1.42) | 0.03 | 0 |
|  | PC aa C42:1 | 0.32±0.08 | 0.31 | [0.26;0.36] | (0.11 ;0.78) | 0.002 | 0 |
|  | PC aa C42:2 | 0.25±0.07 | 0.24 | [0.20;0.29] | (0.13;0.79) | 0.06 | 0 |
|  | PC aa C42:4 | 0.18±0.04 | 0.18 | [0.15;0.20] | (0.09;0.40) | 0.002 | 0 |
|  | PC aa C42:5 | 0.32±0.11 | 0.30 | [0.26;0.36] | (0.14;2.34) | 0.001 | 0 |
|  | PC aa C42:6 | 0.47±0.20 | 0.45 | [0.38;0.53] | (0.20;4.79) | 0.07 | 0 |
|  | PC ae C30:0 | 0.52±0.14 | 0.49 | [0.42;0.59] | (0.24;1.06) | 0.09 | 0 |
|  | PC ae C30:1 | ND | ND | ND | ND | 0.02 | 100 |
|  | PC ae C30:2 | 0.12±0.03 | 0.12 | [0.10 ;0.14] | (0.05;0.25) | 0.01 | 0 |
|  | PC ae C32:1 | 3.08±0.63 | 2.99 | [2.65;3.47] | (1.50;5.61) | 0.001 | 0 |
|  | PC ae C32:2 | 0.79±0.18 | 0.76 | [0.65;0.91] | (0.36;1.53) | 0.007 | 0 |
|  | PC ae C34:0 | 1.68±0.45 | 1.62 | [1.36;1.93] | (0.72;3.60) | 0.009 | 0 |
|  | PC ae C34:1 | 11.5±2.22 | 11.2 | [9.87;12.8] | (6.63;18.7) | 0.02 | 0 |
|  | PC ae C34:2 | 13.3±3.07 | 12.9 | [11.1;14.8] | (6.28;25.7) | 0.001 | 0 |
|  | PC ae C34:3 | 9.39±2.46 | 9.03 | [7.62;10.87] | (4.23;21.23) | 0.002 | 0 |
|  | PC ae C36:0 | 0.79±0.21 | 0.76 | [0.64;0.91] | (0.19;1.77) | 0.05 | 0 |
|  | PC ae C36:1 | 9.75±2.27 | 9.47 | [8.12;11.13] | (4.61;19.26) | 0.03 | 0 |
|  | PC ae C36:2 | 16.8±3.54 | 16.5 | [14.2;18.9] | (7.69;28.6) | 0.03 | 0 |
|  | PC ae C36:3 | 8.59±1.90 | 8.32 | [7.27;9.64] | (4.14;16.78) | 0.001 | 0 |
|  | PC ae C36:4 | 20.7±4.81 | 20.0 | [17.2;23.3] | (10.3;39.4) | 0.02 | 0 |
|  | PC ae C36:5 | 14.5±3.78 | 14.2 | [11.9;16.6] | (6.44;32.2) | 0.005 | 0 |
|  | PC ae C38:0 | 2.55±0.81 | 2.46 | [1.97;2.99] | (0.98;7.94) | 0.08 | 0 |
|  | PC ae C38:1 | 0.80±0.38 | 0.73 | [0.58;0.91] | (0.05;3.52) | 0.002 | 0 |
|  | PC ae C38:2 | 2.00±0.60 | 1.95 | [1.60;2.32] | (0.35;4.67) | 0.003 | 0 |
|  | PC ae C38:3 | 4.46±1.00 | 4.33 | [3.74;5.02] | (2.42;9.80) | 0.005 | 0 |
|  | PC ae C38:4 | 14.9±2.96 | 14.5 | [12.9;16.7] | (7.51;26.5) | 0.01 | 0 |
|  | PC ae C38:5 | 19.3±3.87 | 19.0 | [16.5;21.4] | (11.1;32.5) | 0.005 | 0 |
|  | PC ae C38:6 | 8.90±2.29 | 8.73 | [7.27;10.10] | (3.68;18.53) | 0.001 | 0 |
|  | PC ae C40:1 | 1.45±0.34 | 1.41 | [1.21;1.66] | (0.55;2.71) | 0.006 | 0 |
|  | PC ae C40:2 | 2.18±0.55 | 2.12 | [1.78;2.49] | (0.97;4.68) | 0.005 | 0 |
|  | PC ae C40:3 | 1.16±0.25 | 1.12 | [0.98;1.29] | (0.66;2.73) | 0.002 | 0 |
|  | PC ae C40:4 | 2.40±0.49 | 2.35 | [2.06;2.66] | (1.25;4.37) | 0.04 | 0 |
|  | PC ae C40:5 | 3.74±0.74 | 3.67 | [3.24;4.16] | (1.95;7.49) | 0.002 | 0 |
|  | PC ae C40:6 | 5.40±1.29 | 5.23 | [4.51;6.23] | (2.25;10.45) | 0.002 | 0 |
|  | PC ae C42:0 | ND | ND | ND | (ND;1.92) | 0.48 | 85 |
|  | PC ae C42:1 | 0.34±0.07 | 0.33 | [0.29;0.38] | (ND;0.59) | 0.07 | 1 |
|  | PC ae C42:2 | 0.64±0.14 | 0.62 | [0.54;0.72] | (0.31;1.14) | 0.008 | 0 |
|  | PC ae C42:3 | 0.84±0.18 | 0.81 | [0.71;0.95] | (0.44;1.48) | 0.003 | 0 |
|  | PC ae C42:4 | 0.92±0.21 | 0.90 | [0.77;1.04] | (0.40;1.84) | 0.001 | 0 |
|  | PC ae C42:5 | 2.19±0.44 | 2.16 | [1.90;2.42] | (1.12;4.59) | 0.54 | 0 |
|  | PC ae C44:3 | 0.12±0.03 | 0.11 | [0.10;0.14] | (0.05;0.24) | 0.02 | 0 |
|  | PC ae C44:4 | 0.39±0.09 | 0.38 | [0.33;0.45] | (0.18;0.86) | 0.05 | 0 |
|  | PC ae C44:5 | 1.92±0.45 | 1.88 | [1.62;2.19] | (0.84;4.33) | 0.08 | 0 |
|  | PC ae C44:6 | 1.32±0.32 | 1.29 | [1.09;1.50] | (0.58;2.92) | 0.02 | 0 |

aa: Diacylphosphatidylcholine – ae: Acylalkylphosphatidylcholine

LOD: Limit of detection, ND: Not detected (below LOD)
