# Supplementary material for: The human plasma-metabolome: Reference values in 800 French healthy volunteers; impact of cholesterol, gender and age
Source: PLoS One. 2017 Mar 9;12(3):e0173615. doi: 10.1371/journal.pone.0173615 (PMC5344496; doi:10.1371/journal.pone.0173615)
Supplement: S6 Table — (DOCX) [file pone.0173615.s006.docx]

**S6 Table: Reference values for 5 hydroxysphingomyelins and 10 sphingomyelins**

|  |  | Mean ± SD **(µmol/L)** | Median | Inter-quartile Range | Extreme values | LOD | % ND |
| --- | --- | --- | --- | --- | --- | --- | --- |
|  |  |  |  |  |  |  |  |
| **Sum of sphingomyelin (µmol/L)** | | 288.0±51.1 | 285.3 | [252.8;321.2] | (165.9;447.2) |  |  |
|  |  |  |  |  |  |  |  |
|  | SM (OH) C14:1 | 7.11±1.74 | 6.90 | [5.82;8.16] | (3.27;14.76) | 0.001 | 0 |
|  | SM (OH) C16:1 | 4.10±0.96 | 3.99 | [3.43;4.74] | (2.06;7.06) | 0.001 | 0 |
|  | SM (OH) C22:1 | 15.3±3.55 | 15.1 | [12.8;17.6] | (6.18;28.2) | 0.009 | 0 |
|  | SM (OH) C22:2 | 14.1±3.42 | 13.6 | [11.6;16.2] | (6.84;26.2) | 0.001 | 0 |
|  | SM (OH) C24:1 | 1.56±0.40 | 1.51 | [1.26;1.80] | (0.70;3.57) | 0.001 | 0 |
|  | SM C16:0 | 114.4±19.7 | 113.9 | [100.2;126.2] | (70.1;178.7) | 0.01 | 0 |
|  | SM C16:1 | 16.6±3.36 | 16.3 | [14.0;18.6] | (8.33;31.6) | 0.01 | 0 |
|  | SM C18:0 | 23.8±5.28 | 23.4 | [20.1;26.7] | (10.5;46.2) | 0.01 | 0 |
|  | SM C18:1 | 11.0±2.56 | 10.7 | [9.23;12.6] | (5.07;23.9) | 0.001 | 0 |
|  | SM C20:2 | 0.35±0.11 | 0.34 | [0.27;0.41] | (0.07;0.94) | 0.001 | 0 |
|  | SM C22:3 | ND | ND | ND | ND | 0.001 | 100 |
|  | SM C24:0 | 22.0±4.73 | 21.7 | [18.7;24.8] | (9.01;45.3) | 0.07 | 0 |
|  | SM C24:1 | 57.1±11.7 | 56.2 | [48.8;63.7] | (28.4;104.0) | 0.01 | 0 |
|  | SM C26:0 | 0.23±0.07 | 0.23 | [0.19;0.27] | (0.07;0.57) | 0.001 | 0 |
|  | SM C26:1 | 0.47±0.13 | 0.46 | [0.38;0.54] | (0.20;1.18) | 0.001 | 0 |

LOD: Limit of detection, ND: Not detected (below LOD)
